# The effects of physical activity and exercise therapy on frail elderly depression: A narrative review

**DOI:** 10.1097/MD.0000000000034908

**Published:** 2023-08-25

**Authors:** Yaqun Zhang, Xin Jiang

**Affiliations:** a School of Sports Science, Anshan Normal University, Anshan, China; b School of Physical Education, Dalian University, Dalian, China.

**Keywords:** depression, effects, exercise therapy, frail elderly, narrative review, physical activity

## Abstract

Due to aging, decreased immune function, and an increase in various diseases, frail elderly people are prone to symptoms of depression, which may seriously affect their quality of life. Physical activity and exercise therapy have been identified as a promising method for preventing and treating depression in the elderly. This narrative review assesses the relationship between physical activity and depressive symptoms in frail elderly people, the mechanisms by which physical activity affects depressive symptoms, and the effectiveness of exercise therapy on the health status of frail elderly people. Through searches on the web of science, scopus, PubMed, and CNKI databases, there are a large number of studies on the relationship between physical activity and depression; However, few of them give us a mechanism for how physical activity affects depression. Although all progress has been made in developing appropriate exercise therapy to treat depression, the mechanisms underlying the effects of different types of exercise therapy on depression in frail elderly people have not been satisfactorily addressed, and the optimal effect of exercise therapy on depression cannot be achieved. In this way, future research should more effectively clarify the mechanism of physical activity affecting depression in frail elderly people in China, in order to understand which exercise therapy or how to formulate exercise prescriptions can make exercise therapy play the greatest role in treating depression in vulnerable elderly people in China.

## 1. Introduction

By the end of 2018, China's elderly population aged 60 and above was approximately 249 million, and the population aged 65 and above was approximately 167 million. However, the overall health status of the elderly in China is not optimistic. More than 180 million elderly people are in a frail state, and they suffer from chronic diseases, with a proportion of 75% suffering from one or more chronic diseases.^[1]^ The frail elderly are considered to be a group of people with increased vulnerability caused by degenerative changes in the body and a variety of chronic diseases. The health characteristics of the frail elderly are the decline of their physiological functions or the abnormality of their multi-system functions, and the risk of negative clinical events in the elderly is increased. The problem of the frail elderly is considered to be one of the problems facing global aging. Due to aging, decreased immune function and the increase of various diseases, the frail elderly are prone to psychological problems, such as fear, loneliness and depression, which may seriously affect their quality of life.^[2]^ Globally, depression is one of the largest factors causing non-fatal health loss.^[3]^ Elderly depression refers to a mental disorder that first occurs after the age of 60, with persistent depressive mood as the main clinical symptom. The clinical features are mainly low mood, anxiety, delayed thinking and behavior, and various physical discomfort symptoms. China has the largest population of elderly depression, accounting for 7% to 10% of the elderly population.^[4]^ Generally, the disease has a long course and can be cured, but it is easy to relapse. Elderly depression can cause severe insomnia, worsened physical condition, decreased daily living ability, severely impaired quality of life, and even lead to self injury and suicide.^[5]^ The depressive symptoms of the frail elderly have been proven to be related to a series of negative health outcomes, such as cardiovascular disease, dementia, type 2 diabetes, and low levels of physical activity. The incidence rate of depression in women is about twice that in men, and it is related to functional disability, medical conditions of comorbidity and social deprivation.^[6]^ The extension of life expectancy leads to the aging of the population. For the elderly, depression is a major public mental health problem. Research shows that the prevalence of depression in the elderly is relatively high, with a prevalence of 25.1%. In addition, among the elderly, depression is also more likely to be related to suicide.^[7]^

Physical activity is a kind of health behavior that can be changed. Research shows that physical activity has a positive effect on the prevention of depression in the elderly.^[8]^ Promoting physical activity to prevent depression may be cost-effective, and physical activity as an adjunctive treatment can avoid the side effects of antidepressants.^[9]^ Physical activity has been identified as a promising way to prevent and treat depression in the elderly. Physical activity is defined as “body movement caused by skeletal muscle contraction.” Body movements include many specific parameters, such as type, intensity, duration and frequency.^[10]^ A study shows that physical activity can reduce or improve depressive symptoms, which is related to the intensity, time and form of physical activity.^[11]^

In recent years, researchers in Europe and the United States have conducted more in-depth research on the correlation between physical activity and depression of the frail elderly.^[12–14]^ However, the research on physical activity and depression of the frail elderly in China is still in its infancy, and few studies have explored the correlation between them.^[15]^ This narrative review assessed the relationship between physical activity and depressive symptoms in the frail elderly, the mechanism by which physical activity affects depressive symptoms, and the effectiveness of exercise therapy on the health status of the frail elderly. The purpose of this study is to draw the attention of Chinese researchers to the physical activities and depression of the frail elderly, and to provide reference for the establishment of scientific and effective exercise therapy to consolidate the physical and mental health of the frail elderly. Through searches on the databases of web of Science, Scopes, PubMed, and CNKI, there are numerous studies on the relationship between physical activity, depression, exercise and depression, frail elderly people, and physical activity and depression, published from January 1, 2000 to December 31, 2022; reviewed the identified literature. The review follows 4 procedural steps: comprehensive search; time theory/structural evaluation; result synthesis; and critical evaluation. Twenty-eight studies met the inclusion criteria and were included in the review.

## 2. Methods

Through searches on the databases of Web of Science, Scopes, PubMed, and CNKI, there are numerous studies on the relationship between physical activity, depression, exercise and depression, frail elderly people, and physical activity and depression, published from January 1, 2000 to December 31, 2022; reviewed the identified literature. The review follows 4 procedural steps: comprehensive search; time theory/structural evaluation; result synthesis; and critical evaluation. Twenty-eight studies met the inclusion criteria and were included in the review.

### 2.1. Definition and diagnosis of frailty

Dr Fried put forward in 2001 that frailty is a clinical syndrome characterized by weakened physiological reserve function and multi-system disorder, which reduces the body's ability to stress and maintain internal environment stability, and increases its susceptibility to stress events.^[16]^ Rockwood believes that frailty is a dangerous state caused by the accumulation of health defects.^[17]^ The diagnosis of frailty lacks a unified standard.^[18]^

### 2.2. Effect of different physical activity levels on improving depression in frail elderly

Regular physical activities can improve the depression symptoms of the elderly, and physical activities of different intensities have certain effects on improving the depression symptoms of the elderly.^[19–21]^ A study suggests that sedentary behavior for a long time will increase the risk of depression in the elderly. Regular physical exercise can reduce the risk of depression in the elderly, and the risk of depression in the elderly who lack physical exercise is higher than that in the elderly who regularly exercise.^[22]^ A study found that compared with low-intensity physical exercise, high-intensity physical exercise has a positive effect on reducing depressive symptoms.^[23]^ Another study also found that high-intensity resistance training has a more significant effect on improving depression in the elderly than low-intensity resistance training.^[24]^ However, some studies have shown that both low-intensity physical activity and medium-high-intensity physical activity can significantly improve the symptoms of the elderly and reduce the risk of depression in the elderly.^[25]^ A study showed that compared with high-intensity physical exercise, medium-low-intensity physical exercise has the effect of protecting the elderly from depression. The study also believed that exercise of increasing flexibility can also reduce the risk of depression in the elderly to a certain extent, such as yoga, Pilates, etc.^[26]^

A study has proved that the amount of physical activity has different effects on improving adult depression. With the increase of physical activity, the degree of depression and anxiety of individuals are also decreasing, which indicates that the amount of physical activity is negatively correlated with the degree of depression.^[27]^ Bernard^[28]^ found that there is a dose relationship between the level of physical activity and mental health of adults, but whether there is a dose relationship between the level of physical activity and mental health of the elderly also needs to be explored by a large number of experiments. The study found that the most common treatment for depression in the elderly is drug treatment and psychotherapy.^[29]^ The disadvantage of drug therapy and psychotherapy is that they need a good economic foundation. People with poor family conditions cannot get good treatment. Moreover, long-term medication will also have a bad impact on human functions, such as drug obesity. So researchers should look for more cost-effective interventions to solve the problem of treating depression. Researchers^[30]^ conducted a study to compare the effectiveness of exercise intervention and antidepressant drugs in alleviating depression in the elderly. The study results showed that exercise intervention had good results in alleviating depression in the elderly. After drug treatment, depression in the elderly also improved, but the improvement was less significant than that in the exercise intervention group. Researchers^[31]^ conducted a study to verify the efficacy of aerobic exercise and sertraline (an antidepressant) in the treatment of severe and mild depression in the elderly. The results showed that there was no difference between the efficacy of aerobic exercise in the treatment of severe and mild depression in the elderly and the efficacy of sertraline. Researchers^[32]^ conducted a study to verify the difference between the effects of drug therapy alone and Taijiquan combined with drug therapy on depression in the elderly. The results showed that compared with the use of antidepressants alone, Taijiquan combined with antidepressants has a more significant effect on depression in the elderly.

There is a dose relationship between the level of physical activity and mental health in adults, that is, high-intensity physical activity is negatively correlated with depressive symptoms. However, whether there is a dose relationship between the level of physical activity and mental health in the elderly requires extensive experimental research to explore. Currently, it can be determined that both exercise and exercise combined drug therapy can significantly improve the depressive mood of the elderly, but whether specific exercise therapy has better effects or whether antidepressant drug therapy has better effects still needs to be further verified through experimental research in the future.

### 2.3. Effects of different types of physical activities on improving depression in the elderly

A study shows that various types of exercise can play a certain role in the treatment of depression in the elderly,^[33]^ such as aerobic exercise, resistance training, Tai Chi, Qigong, yoga, and aerobic and anti-resistance mixed exercise. It is meaningful to explore which type of physical activity is more effective in alleviating and treating depression in the elderly. A study showed that^[34]^ aerobic exercise can relieve anxiety and depression symptoms of the disabled elderly. A study shows that,^[35]^ Taijiquan can effectively relieve the depression symptoms of the elderly. A meta-analysis shows that^[36]^ also proves that Taijiquan has a more obvious effect on relieving depression symptoms in the elderly. A study^[37]^ using sitting yoga can reduce the depression symptoms of the elderly, and compared with the non-yoga group, yoga has a more significant effect on reducing the depression scores of the elderly women, but compared with the aerobic exercise group, the yoga group has no significant effect on reducing the depression scores. At present, there is no clear mechanism that different types of physical activities have different effects on depression in the elderly, so more research is needed to distinguish the effects of different types of physical activities on alleviating and treating depression in the elderly.

### 2.4. Mechanisms related to physical activity and depression in frail elderly

Several possible mechanisms of the relationship between physical activity and depression have been proposed and tested. Physical activity may improve the physical and psychological conditions of the elderly to prevent and improve depression.

### 2.5. Structural changes of brain

Changes in the brain structure of the elderly may be one of the mechanisms leading to depression in the elderly, such as structural changes in the prefrontal cortex, anterior cingulate cortex, hippocampus, and corpus callosum.^[38]^ These changes in brain structure also have a significant impact on the cognitive state of older people. Moderate intensity exercise can change the control of the nervous system, regulate central nervous excitation levels, improve brain plasticity in the elderly, improve their metabolism, release dopamine, and improve negative emotions, such as depression and anxiety.^[39]^ Researchers analyzed brain magnetic resonance imaging data from patients with depression and found that those who suffered from repeated episodes of depression also suffered from symptoms of reduced hippocampal size.^[40]^ A study has shown that there are problems with the interaction and communication between the prefrontal cortex and the limbic system in the brain when depression occurs. The prefrontal cortex is basically the part of the brain responsible for thinking, while the limbic system is the part responsible for feeling. The prefrontal cortex, which is responsible for thinking, should have helped regulate the limbic system, which is responsible for sensation. However, in a depressive state, the interaction and communication patterns of these brain regions deviate from the right track.^[41]^

### 2.6. Damage of nerve cells

A study showed that,^[42]^ depression may be related to the damage of hippocampal neurons and other neurons directly involved in stress regulation. Physical activity can enhance the content of blood oxygen, the nutrient factors of hippocampal neurons and stress-regulated cells. These nutrient factors can improve the metabolism of the brain, nourish the cells of the nervous system, and then repair the damaged hippocampal neurons and other cells directly involved in stress regulation.^[43]^

### 2.7. Decrease of cardiovascular system function

A study shows that,^[44]^ compared with the elderly with normal cardiovascular system function, the elderly with cardiovascular diseases are more prone to depression. Physical activity can alleviate depression by improving the cardiovascular system function of the elderly. However, the mechanism of the relationship between cardiovascular system function and depression in the elderly is still unclear.

### 2.8. Increase of inflammatory factors

A study showed that,^[45]^ the test results of physiological and biochemical indicators of patients with depression showed that the level of inflammatory markers in patients with depression was higher than normal value, such as interleukin-10, interleukin-6, tumor necrosis factor and C-reactive protein. Another study showed that^[46]^ physical activity can create an anti-inflammatory environment in the human body. During physical activities, the human body will stimulate the hypothalamus-pituitary-adrenal axis to promote the release of adrenal hormones, cortisol and anti-inflammatory cytokine-interleukin-10, which can reduce the release of pro-inflammatory cytokines in the hippocampus. Physical activity can improve the inflammatory environment of the internal environment by promoting the release of anti-inflammatory cytokines.^[47]^ Both antidepressant drugs and physical activities can create an anti-inflammatory environment in the internal environment of the human body, which indicates that physical activities can be used as a way to replace drugs to treat depression.

### 2.9. Effect of aerobic exercise on depression in the frail elderly

Researchers conducted a randomized controlled trial to study the impact of aerobic exercise on the treatment of frail elderly patients with severe depression. A total of 156 frail elderly people with an average age of 67 years participated in the trial, and were then divided into an aerobic exercise treatment group and a control group (without any intervention). The randomized controlled trial lasted 10 months. Research results show that aerobic exercise can significantly improve the depressive mood of elderly patients with depression. There is no significant difference between the efficacy of aerobic exercise and antidepressants, and aerobic exercise can be used instead of medication.^[48]^ Regular walking is one of the most recommended and popular sports activities in the world. Orcin et al^[49]^ conducted a study to verify the impact of unsupervised regular walking on physical function, cognitive function, emotional state, and quality of life in middle-aged and elderly frail elderly people. A total of 40 middle-aged and elderly frail elderly people participated in the study as an experimental group, with an average age of 56.30 ± 4.85 years. Walking learning lasted for a year. The subjects walked at least 3 times a week and at least 45 minutes a day. The control group also included 40 subjects. The control group did not perform regular walking exercises. The average age of participants in the control group was 55.15 ± 5.64 years old. Before the test, participants’ sociodemographic data were recorded, and their body mass index and waist to hip ratio were calculated. The physical functions and qualities (grip strength, balance ability, cardiovascular endurance, flexibility, muscle endurance, and coordination), cognitive function, emotional status, health status, and quality of life of the 2 groups of frail elderly were evaluated. The results showed that after 1 year of experiment, there were significant differences in all measurement results between the experimental group and the control group (*P* < .05). The improvement of most parameters in the conventional walking group was higher than that in the inactive control group, and the depression score in the conventional walking group was lower than that in the control group. Unsupervised regular walking can significantly reduce the depression score of frail elderly people, and is also a safe, inexpensive, and easy exercise method to adapt to their daily lives. Research by Cukier^[50]^ has shown that aerobic exercise has many beneficial effects on mental health, including reducing anxiety and depression and improving people's happiness. Researchers conducted a study to investigate the effects of high-intensity exercise, low-intensity aerobic exercise, and inactivity on anxiety, depression, and health in frail young and elderly people. The results showed that high-intensity exercise had a positive impact on the mental health of frail young participants, but it was not suitable for the elderly; low-intensity aerobic exercise can improve the mental health of frail elderly people, such as improving anxiety and depression and increasing the happiness of frail elderly people. Low-intensity aerobic exercise is a suitable exercise method for frail elderly people, which can improve their mental health. Aerobic exercise has a positive effect on improving the depressive mood of frail elderly people, but which type of aerobic exercise has a more significant effect on improving the depressive mood of frail elderly people is relatively less effective. Comparative studies are needed to verify which aerobic exercise is more effective in improving depression in frail elderly people.

### 2.10. Effect of resistance exercise on depression in the frail elderly

Tapps TN^[51]^ conducted a study to determine the impact of 12-week resistance exercise on depression levels in frail elderly people. A total of 40 frail elderly people participated in the study and were randomly assigned to the resistance exercise group and the control group. During the implementation of the study, each frail elderly person should complete the Becker Depression Scale at the beginning of the experiment, the 4th, 8th, and 12th weeks to measure the level of depression in frail elderly people. The results showed that compared with the control group, the level of depression in the frail elderly in the resistance exercise group was lower, indicating that resistance exercise can positively improve the depressive status of the frail elderly. A meta-analysis^[52]^ shows that resistance exercise can actively improve the level of depression and anxiety symptoms and quality of life of the frail elderly with type 2 diabetes, while aerobic exercise does not change the level of depression and anxiety symptoms and quality of life of the frail elderly with type 2 diabetes. Castaneda Sceppa C^[53]^ conducted a study to determine the impact of a 16 week high-intensity progressive resistance exercise training program on the mental health of the frail elderly with type 2 diabetes. A total of 58 elderly Puerto Ricans participated in the study and were randomly assigned to a supervised resistance exercise training group (n = 29) or a control group (n = 29). At the beginning of the experiment and at the end of the 16-week experiment, 2 mental health assessment scales were used to assess the depression status (depression scale) and quality of life (short from health survey 36) of the frail elderly with type 2 diabetes. At the beginning of the experiment, there was no difference in mental health measurements between the 2 groups of frail elderly people. After 16 weeks of experiment, the scores of depression and quality of life of the elderly with type 2 diabetes and infirmity in the resistance exercise training group were significantly improved. The study concluded that it is feasible to incorporate resistance exercise training programs into the treatment of mental health disorders of the frail elderly with type 2 diabetes, but researchers need to do more research to understand the mechanism of this situation and the feasibility of applying resistance exercise training programs to the elderly community. Jess et al^[54]^ conducted a study to determine the impact of supervised physical exercise and antidepressant therapy on frail elderly patients with depression. The study lasted 6 months. A total of 312 frail elderly depression patients over the age of 65 were randomly assigned to either the supervised resistance exercise program group or the antidepressant drug group. Participants’ physical condition will be evaluated at the beginning of the experiment and reevaluated at 15 days and 1, 3, and 6 months. The supervised exercise program will include 2 sessions per week with 10 to 12 patients per group for a period of 6 months. Sports coaches train patients for at least 30 minutes of resistance training almost every day. The results showed that the depression scale scores (Montgomery Asberg Depression Scale and Elderly Depression Scale) of frail elderly patients with depression were significantly lower than those before the experiment. The depression symptom score of frail elderly depression patients in the drug treatment group also significantly decreased, indicating that resistance exercise and antidepressant drugs have the same impact on depression symptoms in frail elderly depression patients.

Currently, there is evidence that resistance exercise is beneficial for frail elderly patients with depression and may be equivalent to antidepressant therapy, but the best method for implementing this recommendation in clinical practice is unclear.

### 2.11. Effect of Chinese traditional physical exercise on depression in the frail elderly

As a way to maintain health in China, Taijiquan has received increasing attention in China, Asia, and the West.^[55]^ In China, most people practice tai chi in the park every morning. Taijiquan can more effectively improve the mental health of frail elderly people and improve their depression and anxiety.^[56]^ Nearly 2-thirds of frail elderly patients receiving medication to treat depression fail to achieve remission of symptoms of depression. Lavretsky H et al^[32]^ added Taijiquan exercise to the drug treatment of depressive symptoms in frail elderly patients with depression to verify whether Taijiquan combined with drug treatment can enhance the therapeutic effect of depression in frail elderly patients, aiming to alleviate depressive symptoms in frail elderly patients and improve cognitive ability. A total of 112 frail elderly people aged 60 and over with severe depression participated in the study and received approximately 4 weeks of antidepressant treatment. Seventy-three frail elderly patients with partial reactions to antidepressants continued to receive etalopram daily and were randomly assigned to 10 weeks of combined adjuvant therapy: 2 hours of tai chi per week; 2 hours of health education per week. All participants were evaluated for depression, anxiety, quality of life, cognition, and inflammation during pre-experiment and 14-week follow-up. The results showed that subjects who received antidepressant drugs combined with Tai Chi boxing were more likely to exhibit greater depression symptom relief and depression relief than subjects who received antidepressant drugs combined with health education. Research has concluded that the combination of assisted depression treatment methods such as Taijiquan may further improve the clinical efficacy of medication in the treatment of frail elderly depression.

Taijiquan is a traditional Chinese sport that has beneficial effects on physical, mental, and cognitive health. Taijiquan is also particularly popular in other countries. Researchers^[57]^ from Mexico conducted a study to explore the effects of Tai Chi Boxing on the mental health of weak female practitioners and sedentary disabled women. Before and after the experiment, frail women in the Taijiquan group and the sedentary group were required to receive neuropsychological tests, the Becker Depression and Anxiety Scale, and an assessment of elderly people activities of daily living. The results showed that the levels of depression and anxiety in the weak female group who practiced Tai Chi significantly decreased, while the sedentary group had no significant changes. In the future, it is necessary to understand which functions of frail elderly people can benefit from Tai Chi exercise, which can promote Tai Chi as an alternative treatment for neuropsychological interventions and be used in patients who are frail due to normal or pathological aging.

Although there is sufficient evidence to prove that physical exercise is beneficial to health, only a few frail elderly people are willing to participate in physical exercise. Therefore, there is an urgent need to develop theoretical and evidence-based sports interventions to effectively promote physical activity among the frail elderly.

## 3. Conclusion and limitations

Depression and frail are common problems in the elderly, and physical activity is closely related to their occurrence and prevention. Through reviewing the literature, this study analyzed the correlation between physical activity and depression in the frail elderly, which has important theoretical guiding significance for preventing and improving depression in the frail elderly. However, the research on physical activity and depression of the frail elderly in China is at the initial exploration stage, and there is not enough clinical evidence to provide guidance and suggestions for exercise intervention to treat the depression of the frail elderly. In the future, exercise intervention can be carried out for the frail elderly based on the correlation between physical activity and depression to comprehensively promote the physical and mental health of the frail elderly, improve the degree of frailty, and improve the quality of life of the frail elderly. Therefore, according to the frailty degree and health characteristics of the frail elderly, building a sports health promotion system is the focus of future research on the physical and mental health promotion of the frail elderly.

Our narrative review is also influenced by many limitations. Firstly, there are relatively few databases searched for keywords in this narrative review, and in the future, more databases need to be searched to increase the reliability of this narrative review. Secondly, in the narrative review, we found a relationship between the level of physical activity in frail elderly people and depressive symptoms, as well as the effectiveness of exercise therapy. In future research, the relationship between physical activity and depression and the effectiveness of exercise therapy can be observed by categorizing according to different ethnic groups. Thirdly, the research included in this narrative review was published from January 1, 2000 to December 31, 2022, which may affect the reliability of the results.

## Author contributions

**Investigation:** Yaqun Zhang, Xin Jiang.

**Methodology:** Yaqun Zhang, Xin Jiang.

**Project administration:** Yaqun Zhang, Xin Jiang.

**Writing – original draft:** Yaqun Zhang, Xin Jiang.

**Writing – review & editing:** Yaqun Zhang, Xin Jiang.
